# A Distance-Vector-Based Multi-Path Routing Scheme for Static-Node-Assisted Vehicular Networks

**DOI:** 10.3390/s19122688

**Published:** 2019-06-14

**Authors:** Daichi Araki, Takuya Yoshihiro

**Affiliations:** 1 Graduate School of Systems Engineering, Wakayama University, 930 Sakaedani, Wakayama 640-8510, Japan; araki.daichi@g.wakayama-u.jp; 2 Faculty of Systems Engineering, Wakayama University, 930 Sakaedani, Wakayama 640-8510, Japan

**Keywords:** vehicular networks, delay tolerant networks, distance-vector routing

## Abstract

Vehicular Ad hoc NETworks (VANET) has been well studied for a long time as a means to exchange information among moving vehicles. As vehicular networks do not always have connected paths, vehicular networks can be regarded as a kind of delay-tolerant networks (DTNs) when the density of vehicles is not high enough. In this case, packet delivery ratio degrades significantly so that reliability of networks as an information infrastructure is hardly held. Past studies such as SADV (Static-node Assisted Data dissemination protocol for Vehicular networks) and RDV (Reliable Distance-Vector routing) showed that the assistance of low-cost unwired static nodes located at intersections, which work as routers to provide distance-vector or link-state routing functions, significantly improves the communication performance. However, they still have problems: SADV does not provide high-enough delivery ratio and RDV suffers from traffic concentration on the shortest paths. In this paper, we propose MP-RDV (Multi-Path RDV) by extending RDV with multiple paths utilization to improve performance against both of those problems. In addition, we apply a delay routing metric, which is one of the major metrics in this field, to RDV to compare performance with the traffic-volume metric, which is a built-in metric of RDV. Evaluation results show that MP-RDV achieves high load-balancing performance, larger network capacity, lower delivery delay, and higher fault tolerance against topology changes compared to RDV. As for routing metrics, we showed that the traffic-volume metric is better than the delay one in RDV because delay measurement is less stable against traffic fluctuation.

## 1. Introduction

Vehicles are regarded as a kind of mobile sensor nodes and vehicular ad hoc networks are regarded as a potential infrastructure to help collecting or delivering various data that may enhance our life. Vehicular Ad hoc NETworks (VANETs) have been deeply studied to realize the dependable data infrastructure that consists of vehicles by means of multi-hop packet forwarding. Although VANETs are expected to support both metropolitan and rural scenarios, especially rural (or suburban) scenarios in which vehicles are not dense enough have a significant problem on packet delivery ratio. If vehicle density is low, VANET is regarded as a kind of Delay-Tolerant Networks (DTNs), and the past contributions on DTN routing have proved that high packet delivery ratio is hardly achievable with sparsely located nodes; with simple schemes such as the epidemic routing or the spray-and-wait approach, a significant load for duplicated packets is required to improve delivery ratio of packets [1,2,3].

A large number of studies have been dedicated to overcoming this sparsity problem in VANET [4,5,6,7,8,9,10,11]. From the early stage of VANET, geographic routing is regarded as one of the best-suited routing strategies, and several techniques have been developed based on it. In DTN-based vehicular networks, vehicle selection to pass packets is a key decision to improve packet delivery ratio. Thus, *carry-and-forward* behavior [8] that affords vehicles to have time to choose the next-hop vehicle has been deployed with techniques for selecting good next-hop vehicles such as utilizing vehicles’ trajectories (e.g., [9]) or estimating vehicle traffic amount of roads (e.g., [10]). However, although those techniques certainly improve the delivery ratio of packets in sparse scenarios of VANETs, the ratio is still too low in order to support practical applications over VANETs.

The idea to utilize static, but not wire-connected wireless nodes located at intersections is introduced in SADV [12]. In SADV, static nodes are located at each intersection and they work as a link-state router; they retrieve the network topology from the roadmap, measure the packet forwarding delay of each link between two neighboring static nodes, and compute the shortest-path to each destination. At intersections, static nodes receive packets from vehicles and pass them to other vehicles that are likely to carry packets along the shortest paths to the destinations. SADV shows that inexpensive unwired static nodes located at each intersection can significantly improve the packet reachability to their destinations. In addition, by forwarding duplicated copies of a packet to the primary and the secondary next-hops at every relay node, i.e., by using multiple paths, SADV further improved the packet delivery ratio. However, since SADV has no specific mechanism to control the expected ratio on end-to-end packet delivery, there are cases in which packet delivery ratio is not sufficiently high, which is inconvenient in practice as a reliable infrastructure. In addition, since every node always forwards packets to the two next-hops, the overhead of communications is high so that the network capacity is limited.

Yoshihiro et al. proposed a distance-vector-based routing scheme RDV that provides the pre-configured expectation on packet delivery ratio by creating the sufficient number of packet copies at static nodes [13]. Namely, if administrators configure to expect 99% end-to-end packet delivery, RDV provides the 99% expected packet delivery ratio. Since RDV creates the minimum number of packet copies to achieve the preconfigured expectation, RDV requires the lowest overhead of duplicated packets. However, since RDV uses a single shortest path to forward packets to a destination, data traffic easily concentrates on the shortest paths and excesses the capacity. In addition, since the paths are selected based on vehicle density rather than delivery delay, the forwarding paths provide far larger delay compared to SADV.

Now note that, although RDV has a strong characteristic providing probabilistic expectation on packet delivery ratio, the performance of RDV has not still been optimized. SADV has two major advantages on RDV that may further improve performance of RDV, that is, multi-path utilization and delay routing metric. Thus, in this paper, as an extension of a conference paper [14], we try to introduce those two features into RDV to improve the communication performance. First, we incorporate the multi-path utilization into RDV to relax the concentration of packets on the shortest paths so as to expand the traffic capacity of RDV. Second, we compare two major routing metric criteria, i.e., traffic volume and delivery delay, in RDV routing schemes in order to make better selection of the metrics.

Specifically, in this paper, we propose MP-RDV (Multi-Path RDV) that extends RDV to utilize multiple paths. MP-RDV forks forwarding paths at each intermediate static node and creates duplicated packet copies for each of the paths to improve the load balancing performance as well as the delivery delay. Inheriting the property of RDV, MP-RDV minimizes the total traffic loads to achieve the preconfigured delivery ratio by computing the minimum number of packet copies to forward on each of the forked paths. In addition, we designed a delay routing metric and its computational mechanism that work with RDV to provide an option of delay-based metric utilization in RDV. Since the delivery delay of packets consists of waiting delay on nodes and transportation delay between nodes, those two values are measured separately in cooperation with related nodes and summed up to compute the delay metric values, and the metric values are advertised by the distance-vector messages.

The simulation results show that MP-RDV provides well load-balanced communications so that larger traffic than both RDV and SADV can be forwarded while simultaneously holding the preconfigured expected delivery ratio. In addition, by utilizing multiple paths simultaneously, delay performance is reduced as the same level as SADV. As for comparison of metrics, we found that the built-in traffic-volume metric has totally better performance because the delay metric is found less stable and reliable against fluctuation of vehicular traffic. The results lead to the conclusion that MP-RDV with traffic-volume metric has totally better communication performance than the conventional methods in the literature.

The reminder of this paper is organized as follows. In Section 2, we describe the related work in the literature and clarify the contribution of this study. In Section 3, we introduce the distance-vector routing scheme RDV, which is the base single-path scheme that we extend in this paper. In Section 4, we present the proposed scheme MP-RDV in detail, and introduce the delay routing metric to MP-RDV. Evaluation is done through simulation in Section 5, which examines the performance of MP-RDV in terms of several measurement criteria. Finally, in Section 6, we conclude the work.

## 2. Related Work

### 2.1. Vehicular Ad Hoc Networks

Since vehicular networks could be a data infrastructure in both urban and rural areas, multi-hop vehicular ad hoc networks have been deeply studied so far. Once geographic routing has been found to be suitable in vehicular networks, several effective techniques have been developed based on geographic routing. Lochert et al. proposed a DTN routing scheme called GPCR (Greedy Perimeter Coordinator Routing) and showed that the geometric routing is one of the suitable choices in routing strategies in VANET [4]. Lee et al. proposed GPSRJ+ (Greedy Perimeter Stateless Routing Junction+) that improves the road segment selection method based on geographic routing to achieve higher packet delivery ratio [5]. Lochert et al. presented GSR (Geographical Source Routing) that introduces an anchor-based routing, in which Dynamic Source Routing (DSR) based on anchors on map is applied into vehicular networks [6]. Seet et al. proposed an anchor-based routing called A-STAR (Anchor based Street and Traffic Aware Routing) that considers streets on maps and vehicular traffic amount on streets in making routing decisions [7]. Zhao et al. proposed VADD (Vehicle-Assisted Date Delivery) in which they introduced the *carry-and-forward* behavior into geometric routing that holds packets until they find adequate next-hop vehicles that are likely to forward packets to the destinations [8]. Wu et al. proposed MDDV (Mobility-centric Data Dissemination Algorithm for Vehicular Networks) that forwards packets along the trajectory precomputed from static road information [9]. Jerbi et al. proposed GyTAR (Greedy Traffic Aware Routing), which considers dynamic traffic information such as density of vehicles to select delay-optimal outgoing roads at each intersection [10]. Zhang et al. proposed a street-centric routing that introduces the concept of micro topologies to consider the wireless connections among vehicles on each street [11]. However, although those techniques certainly improve the delivery ratio of packets in sparse scenarios of VANETs, the delivery ratio is still too low in order to support practical applications over VANETs.

### 2.2. Deployment of Static Nodes in Vehicular Networks

Static nodes have been considered as useful devices to support communications of vehicles. A typical style of static node is called RSU (Road-Side Unit), which often provides single-hop communication with vehicles. The well-known example of such systems is DSRC (Dedicated Short Range Communications), which has already been standardized as IEEE1609 families [15], and also have practically deployed all over the world such as ERP (Electric Road Pricing) in Singapore [16] and VICS (Vehicle Information and Communication Systems) in Japan [17].

RSU also works in combination with multi-hop vehicular networks. To enhance the coverage of RSU, Jeong et al. proposed TSF (Trajectory-based Statistical Forwarding) [18] that delivers packets from RSUs to a target vehicle via multi-hop communications of vehicles. Reversely, Skordylis et al. and Jarupan et al. respectively proposed D-Greedy (Delay-bounded Greedy forwarding) [19] and PROMPT (cross-layer Positionbased coMmunication PROTcol for delay-aware vehicular access networks) [20], which forward packets from vehicles to RSUs via multi-hop communications. Wu et al. regard the problem of packet delivery among vehicles supported by wired RSUs as an optimization problem and solved the problem to provide better performance on packet delivery [21]. O’Driscoll et al. proposed a geographic routing protocol called IEGRP (Infrastructure Enhanced Geographic Routing Protocol) that is enhanced by wired RSUs located at intersections to exchange packets among vehicles [22]. However, they all assume that RSUs have wired connection to networks, which requires high building costs.

On the other hand, Li et al. proposed an approximated algorithm to compute the optimal locations of wired/wireless static nodes in the roadmap that supports delay-bounded propagation of information in VANETs [23]. This study tries to reduce the building cost of RSUs while maximizing the effect on enhancing the performance of vehicular ad hoc networks.

### 2.3. Enhancing Vehicular Ad Hoc Networks with Unwired Static Nodes

Several studies have been made to enhance vehicular ad hoc networks with unwired static nodes. As is already described, SADV [12] is based on link-state routing in which static nodes compute the shortest paths as a sequence of static nodes and build their own routing tables. Each node creates two duplicated copies of a packet and forwards them to the next-hops corresponding to the primary and secondary shortest paths. SADV has no other packet duplication strategy so that it is vulnerable to unexpected packet loss due to vehicles’ movement such as going into narrow streets or parking lots. Note that this effect grows large when static nodes are sparsely located at intersections on the roadmap. Thus, in SADV, static nodes are expected to be installed at a high percentage of intersections.

RDV [13] improved this point by duplicating the required number of packets to suffice the preconfigured expected packet delivery ratio. This not only improves reliability of packet forwarding, but also enables us to make sparse deployment of static nodes located at intersections, i.e., even if we place static nodes at only major intersections between which there is vehicle reach with relatively low probability, packets can be forwarded with a sufficiently high delivery ratio. Sparse deployment reduces deployment cost and makes the routing scheme practical.

ETGR (Efficient Traffic Geographic Routing) [24] is a reactive routing protocol in which the path discovery process using RREQ (Route REQuest) and RREP (Route REPly) packets is assisted by static nodes. ETGR in the original paper treats real-time communications in dense-vehicle scenarios, and compared the communication performance with geometric routing schemes such as GSR [6] and GyTAR [10]. In the sparse scenario, however, due to the dynamic nature of VANET, this approach suffers from large paths repairing cost as well as a large delay to search for available paths to the destinations.

AQRV (Adaptive Quality-of-service (QoS)-based Routing for VANETs) [25] proposed QoS-based routing in static-node-assisted vehicular networks. AQRV assumes urban scenarios in which static nodes are installed at a high percentage of intersections, and computes the communication quality for each road segment between neighboring static nodes. Then, the static nodes behave as routers that dynamically select routes based on an ACO (Ant-Colony Optimization) algorithm. Although AQRV provides high-quality communications with the help of static nodes in urban scenarios with high vehicle density, it does not provide communication quality in sparse scenarios.

EGSR (Efficient GSR) [26] does not explicitly assume stationary devices at intersections, and instead assumes that vehicle density is high enough to hold required information in cooperation with vehicles around intersections. EGSR measures the quality of links (here, a link means the road segment between two adjacent intersections) based on the ACO approach, and keeps sharing the information around intersections. Every vehicle computes the shortest paths using the Dijkstra’s shortest-paths algorithm with the measured link quality as link weight. The weakness of this method, however, appears when it faces sparse cases in which information sharing among vehicles is impossible.

ROSTER (Routing based on SpatioTemporal Encounter Records) [27] utilizes daily trajectory data of vehicles to find good paths with low delay performance in sparse scenarios. By computing the intersection region of trajectories and placing RSUs there, they achieves low delay and high delivery ratio communications in sparse scenarios. Although ROSTER covers majority of communication requirements, they cannot provide full coverage of communication requests in the region because it covers only the major requirements that appear in the trajectory data. In addition, their method highly depends on traffic patterns so that performance could be degraded when traffic pattern changes.

## 3. RDV: The Base Scheme

### 3.1. Overview

RDV assumes that static unwired nodes are sparsely placed at major intersections to help forwarding packets among static nodes. In RDV, static nodes behave as routers and vehicles are the carriers of packets. RDV is a routing scheme that maintains routing tables at each static node in the manner of distance-vector routing scheme. When a vehicle comes into the communication range of a static node, the node exchanges control messages and data packets with the vehicle. Packets are delivered from source static nodes to their destination static nodes via several relay static nodes according to the routing table entries. Since static nodes are located to cover a certain area, RDV supports the packet delivery from and to any locations in the area. Note that, although we describe packet delivery between two static nodes, the sources and the destinations of packets in RDV could not be limited to static nodes but moving vehicles. To support this, we can combine several methods with RDV that provide packet delivery between static nodes (RSUs) and vehicles such as D-Greedy [19], PROMPT [20], or TSF [18].

Vehicles as carriers deliver packets between two static nodes with the *store-and-forward* behavior. When vehicles are in the communication range of static nodes, they switch to *intersection mode*. They pass packets to the node to relay them to other vehicles, and receive packets from the node that should be forwarded toward the vehicle’s on-going direction. Otherwise, when vehicles are not in the communication range of any static node, they work in *road mode*, in which they pass packets to the vehicles ahead of them whenever being possible. In combination of those two modes, RDV provides efficient packet delivery in both urban and rural scenarios.

One of the novel characteristics of RDV is providing reliable packet delivery in that packets are delivered with the pre-configured expected delivery ratio such as 99%. This is done by the function APD (Adaptive Packet Duplication), which creates several duplicated copies of a packet at every relay node to improve the expected delivery ratio to the value pre-configured by the administrators. Note that APD controls the number of duplicated packets to the minimum as long as the expected delivery ratio exceeds the preconfigured value. This significantly reduces the overhead of packet copies and enhances the capacity of networks.

**Figure 1:**
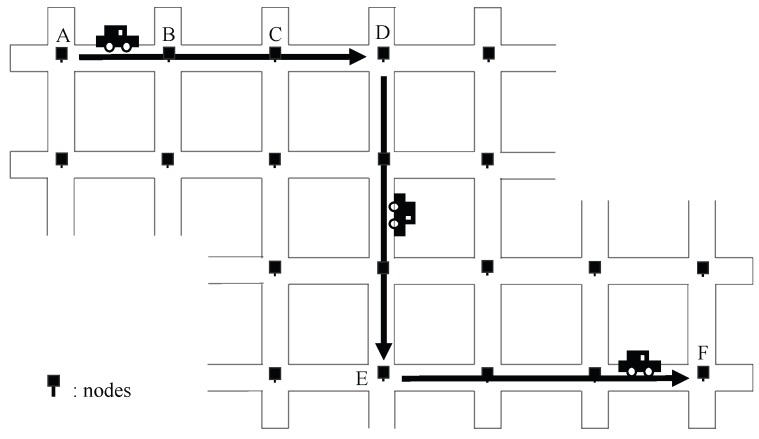
Definitions of Distance Units: The unit *hop* represents the number of static nodes on the path. The unit *carry* represents the number of vehicles required to forward packets on the path.

We define the units in distances specific to RDV as follows. See Figure 1. We define the unit *hop* as the number of static nodes that a path involves. Consequently, since a vehicle carries packets from node A to B without visiting any other static node, the distance from A to B is 1-*hop*. Since a vehicle visits two nodes between A and D, the path is 3-hop long. On the other side, we define the unit *carry* as the number of vehicles required to forward packets. Since a packet can be forwarded from node A to D with a single vehicle, the distance from A to D is 1-*carry* even if it takes several-*hops*. Since a packet is not possible to be forwarded from A to E or F with a single vehicle, the packet must be relayed by static nodes. Since a packet is reachable from A to F via two relay nodes, it must be forwarded by at least three vehicles, so the distance from A to F is 3-*carries*.

### 3.2. Distance-Vector Based Routing Strategy

RDV is designed for vehicular networks to work based on a traditional distance-vector based routing strategy. RDV autonomously detects neighborhoods via control messages to build the shortest-paths so that it does not require map information. Since RDV is independent from maps, RDV is free from maintenance and synchronization cost of map information. Based on the distance-vector routing strategy, nodes in RDV exchange messages periodically to build their routing tables. In the routing table of each static node, a static node in 1-*carry* distance is specified as the *next-relay* for each destination. Since packets are delivered according to routing tables, a packet forwarding path is defined as a chain of static nodes within 1-*carry* distance.

When a node A is going to forward a packet to a 1-*carry* neighbor B, A finds a vehicle that will visit B with high probability, and pass the packet to it. To find a suitable vehicle to pass packets, RDV has a mechanism called SVS (Statistic-based Vehicle Selection), which predicts vehicles that will visit the specified neighbor from the statistical measurements. In SVS, *Hello* messages are sent from each static node to its 1-*hop* and 2-*hop* neighbors by storing a *Hello* message on each vehicle that visits the node. Each node obtains local trajectory information via *Hello* messages. By sharing the information among neighborhoods using *Statistic* messages, every node is ready to compute the best “previous-hop” neighbor node, i.e., the vehicles coming from which will with high probability go to the specified *next-relay* node.

To build a routing table, each node must know the neighbors within 1-*carry* distance. To find 1-*carry* neighbors, RDV uses *Single-carry* messages. Each vehicle distributes the statistical information of SVS with *Single-carry* messages along the roads that the vehicle goes through. As a result, static nodes discover all of their 1-*carry* neighbors, to which packets are delivered with a single vehicle with high probability. The probability of packets to reach a 1-*carry* neighbor is computed as the product of 1-*hop* delivery ratios collected along with the path to the neighbor.

Each node propagates its 1-*carry* neighbors with *Multi-carry* messages in the manner of distance-vector routing. Specifically, a *Multi-carry* message propagates a set of distance-vectors (i.e., basically a set of destinations and the distances for them) to sender’s 1-*carry* nodes. As a result of repeated message propagation, all static nodes finally find the shortest paths for all destinations, and their routing tables have entries for every reachable static node.

With the routing tables computed above, a packet is forwarded to its destination via several intermediate nodes. The next intermediate node for a destination is called *next-relay*, which is retrieved from the routing table at each node. Note that the routing table includes the best *previous-hop* node, the vehicle from which will visit the next-relay with high probability. Thus, when a static node is to forward a packet, it consults with its routing table and find the *previous-hop* node, and passes the packet to the vehicle that comes from the *previous-hop* node. By repeating this forwarding process at each intermediate node, packets finally reach their destinations.

### 3.3. Packet Duplication

In VANET, vehicles as carriers of packets move under their drivers’ own will so that they might go away or park somewhere without forwarding packets to their next-relay nodes. To achieve reliable packet delivery under this kind of uncertainty, RDV creates several copies for each packet and forwards all of them to its next-relay nodes. Note that RDV creates the copies of a packet dedicated to reaching the next-relay node, i.e., the packet copies destine to its next-relay, and if they fail to reach the next-relay, they will be dropped silently. By forwarding multiple copies to a next-relay, we can raise the packet delivery ratio to a pre-configured level even under a relatively high drop probability for each packet.

The mechanism of packet duplication in RDV to achieve the pre-configured expected packet delivery ratio is called APD. In APD, network administrators previously configure the end-to-end packet delivery ratio that they expect, and RDV generates packet copies at each intermediate node so that the expectation of packet delivery ratio exceeds the preconfigured value such as 99%.

Now let *P* be the preconfigured expected delivery ratio to suffice, and let δ be the distance in carry of the path to the destination. (See Table 1 for the definitions.) For each link *l* in the path, we denote the expected packet delivery ratio by $E_{l}$. Since the delivery ratio of the path is expressed by the product of those of the included links, *P* is achieved if each included link *l* suffices ${\left(E_{l}\right)}^{\delta }\geq P$, i.e., the least expected value of $E_{l}$ is expressed as

(1) $$E_{l}=P^{\frac{1}{\delta }}.$$

On the other hand, let $R_{l}$ be the delivery ratio of link *l* measured by SVS, and $D_{l}$ the number of duplicated packet copies on link *l*, respectively. Then, to achieve the delivery ratio $E_{l}$, two variables $R_{l}$ and $D_{l}$ must suffice the following formula:

(2) $$1-{\left(1-R_{l}\right)}^{D_{l}}\geq E_{l}.$$

From this, the smallest value of $D_{l}$ to achieve $E_{l}$ is computed as

(3) $$D_{l}=ceil\left(\frac{log(1-E_{l})}{log(1-R_{l})}\right),$$

where ceil(·) is the ceiling function. RDV achieves the preconfigured expectation of end-to-end delivery ratio if each of source and intermediate nodes generates $D_{l}$ copies according to the above Formula (3).

**Figure 2:**
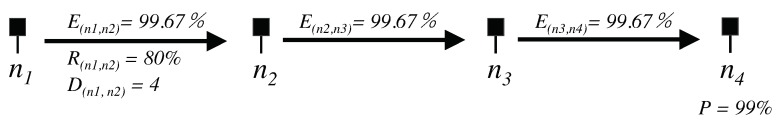
Example of Adaptive Packet Duplication (APD): If the required packet delivery ratio is P=99%, distance is 3-carries, and the packet delivery ratio from $n_{1}$ to $n_{2}$ is 80%, $n_{1}$ creates four duplicated copies of a packet to suffice the delivery ratio *P*.

Figure 2 shows a concise example of APD. The expected delivery ratio that is previously configured by administrators is set as P=99%, and a packet is to be forwarded from $n_{1}$ to $n_{4}$. In this case, $n_{1}$ computes the expected delivery ratio $E_{l}$ for each link $l=(n_{1},n_{2})$, $\left(n_{2},n_{3}\right)$, and $\left(n_{3},n_{4}\right)$ as 99.67% from the above equation. Since the measured delivery ratio of link $\left(n_{1},n_{2}\right)$ derived from SVS is $R_{\left(n_{1},n_{2}\right)}=80\%$, the number of duplicated copies of the packet is computed as $D_{\left(n_{1},n_{2}\right)}=4$ from Formula (3). As a result, $n_{1}$ forwards four packets to $n_{2}$. Note that $E_{l}$ is included in the packet and is notified to $n_{2}$. With this information, $n_{2}$ computes the number of duplicated copies to be forwarded to $n_{3}$ in the same computational process as the case of $n_{1}$.

### 3.4. The Routing Metric

Since RDV is designed to improve delivery ratio, it adopts routing metric by which the paths with large bandwidth, i.e., large vehicle traffic volume, is preferably selected as the packet forwarding paths. To compute the routing metric for each link $\left(n_{s},n_{r}\right)$, RDV computes the expected number of vehicles *V* that go from $n_{s}$ to $n_{r}$ in a unit time using the mechanism of SVS. Then, as customary in the Internet routing to incorporate link bandwidth into the shortest-path computation, RDV defines its routing metric as the inverse of the traffic amount. Namely, the metric $M_{l}^{\left(vol\right)}$ of the link *l* is defined as

(4) $$M_{l}^{\left(vol\right)}=\frac{1}{V}.$$

Note that this value easily fluctuates with time, which leads instability of the forwarding paths. To prevent the fluctuation, it is strongly recommended to apply a smoothing technique such as taking the moving average over time.

### 3.5. An Example

**Figure 3:**
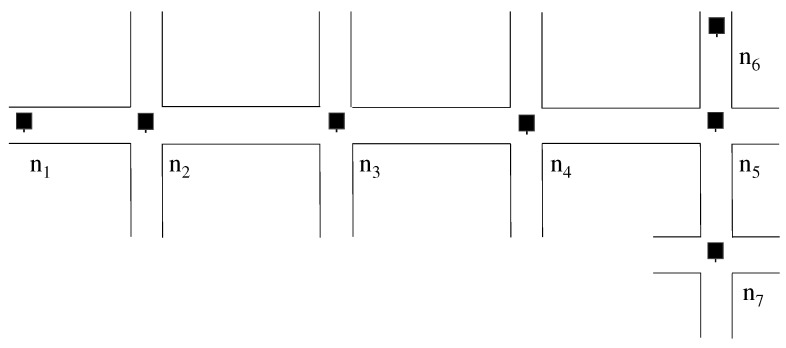
Example Scenario of Packet Forwarding in RDV (Reliable Distance-Vector routing): A packet is to be forwarded from $n_{2}$ to $n_{7}$ with the routing tables of $n_{2}$ and $n_{5}$ shown in Table 2 and Table 3, respectively.

An example scenario of packet forwarding in RDV is depicted in Figure 3 with the routing table of $n_{2}$ and $n_{5}$ shown in Table 2 and Table 3, respectively. Note that each entry of RDV’s routing tables consists of a destination, a next-relay node, a previous-hop node, the distance in *carry* to the destination, the delivery ratio measured by SVS, and the routing metric to the destination. Suppose that a packet is to be forwarded from $n_{2}$ to $n_{7}$. First, $n_{2}$ finds the entry for destination $n_{7}$ and found the corresponding next-relay node is $n_{5}$. Since the previous-hop is $n_{1}$, $n_{2}$ passes the copies of the packet if vehicles from $n_{1}$ visit $n_{2}$. If APD determines to forward multiple copies of the packet, they are passed to different vehicles coming from $n_{1}$. Note that the entry means that, if the packet is passed to the vehicles coming from $n_{1}$, the probability of the packet to reach the next-relay $n_{5}$ is 72%. When $n_{5}$ receives one of the packet copies, $n_{5}$ actually receives the packet. Thus, even if other copies of the same packet are received thereafter, $n_{5}$ silently discards them. Next, $n_{5}$ forwards packets to $n_{7}$ in the same procedure, i.e., $n_{5}$ finds the routing table entry for destination $n_{7}$, passes the packet copies to vehicles coming from $n_{6}$, and finally the destination $n_{7}$ receives the packet. RDV forwards packets in this way to achieve high delivery ratio.

## 4. MP-RDV: The Proposed Scheme

### 4.1. Overview

In this paper, we extend RDV and design MP-RDV in terms of the two drawbacks of RDV. The two drawbacks are the load-balancing and the delivery delay performance described as follows.

**On Load Balancing Performance.** It is observed in [13] that throughput performance is largely degraded when the volume of data traffic exceeds a certain level. This is due to concentration of data traffic on the shortest-path links. Different from SADV that distributes packet copies into the primary and the secondary next-hops, RDV transmits packet copies into the shortest paths only, which makes the load balancing performance degraded. In MP-RDV, we propose to use multiple paths at each node, i.e., we distribute data traffic into primary and the secondary next-relay nodes. With the multi-path extension to RDV, we show that the load-balancing performance as well as the network capacity significantly improve.

**On Delivery Delay Performance.** Simulation results of [13] show that the delay performance of RDV is considerably worse than SADV. This is mainly because of the property of the deployed routing metric. That is, SADV uses the routing metric based on delivery delay, whereas RDV’s metric is designed based on the volume of vehicle traffic on roads. As a result, in exchange for better delivery ratio, RDV’s delay performance is lower than SADV. The countermeasure for this problem is twofold. First, we extend RDV to use multiple paths, which not only improves the load balancing performance but may also improve delivery delay because some of the multiple paths may have smaller delay than the shortest path. Second, we try to make paths selection based on delivery delay. Since delay metric does not work simply in the RDV scheme, we design a mechanism to measure delivery delay of each link in cooperation with related nodes so that the delay metric values are advertised in the distance-vector messages in RDV (as well as MP-RDV).

### 4.2. Selection of Multiple Paths

The basic strategy for multi-paths selection in MP-RDV is to use the primary and the secondary next-relays simultaneously, i.e., each static node forwards the copies of a packets to both relay nodes. However, a naive deployment of this strategy causes several problems.

**Figure 4:**
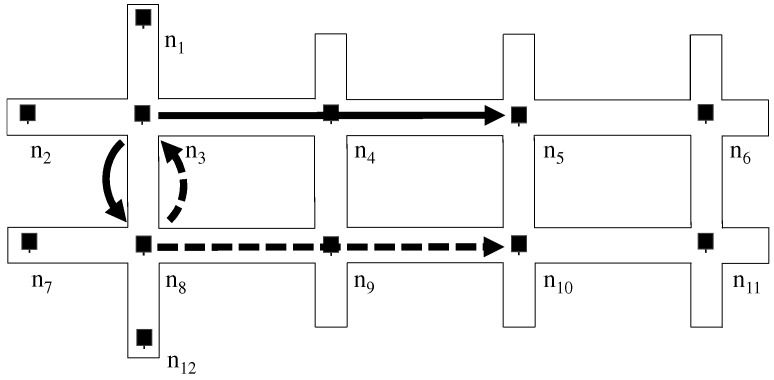
The Routing Loop Problem: When the secondary relay for a destination at $n_{3}$ is $n_{8}$ and the one at $n_{8}$ is $n_{3}$, packets on secondary paths would make routing loops.

The first problem is the so-called routing loop problem, which is shown in Figure 4. In this example, nodes $n_{3}$ and $n_{8}$ have packets that destines to the right side, e.g., $n_{11}$. Node $n_{3}$ has the primary relay $n_{5}$ and the secondary relay $n_{8}$, while $n_{8}$ has the primary relay $n_{10}$ and the secondary $n_{3}$. If $n_{3}$ and $n_{8}$ distribute the packet copies into both relays, packets easily loop between $n_{3}$ and $n_{8}$ that consume the bandwidth and degrades communication performance significantly.

**Figure 5:**
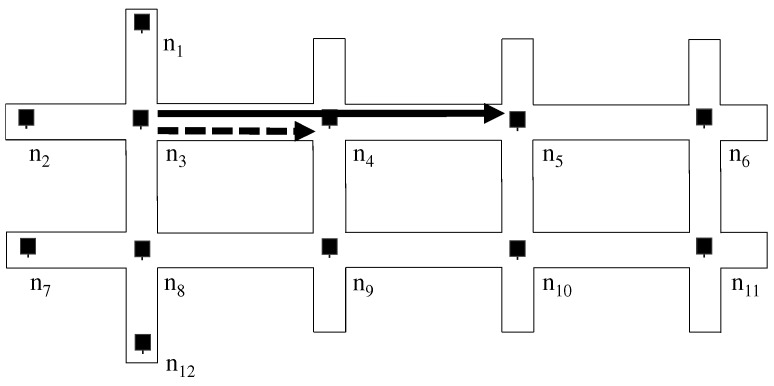
The Same-street Problem: When the primary and secondary relay for a destination at $n_{3}$ is $n_{4}$ and $n_{5}$, respectively, those paths are along the same street and no load balancing effect is expected.

We name the second problem “same-street problem,” which is shown in Figure 5. In this case, suppose that node $n_{3}$ has packets whose destination lies on the right side, e.g., $n_{11}$. If the primary relay is $n_{5}$ and the secondary is $n_{4}$, all packets are going into the same street, defeating the purpose of load balancing.

Unfortunately, both problems easily occur frequently, which makes the multi-path mechanism useless. We must design the method that takes advantage of the merit of multi-path strategy and simultaneously prevents the two harmful defects. To this end, we propose to apply the following two conditions to utilize multiple paths. That is, when a packet is at a static node, only if both of those conditions are met, both the primary and the secondary relays are activated for the packet, and only the primary relay is used otherwise.

(A) The distance in *carry* of the secondary path is the same as or less than the primary path.
(B) The previous-hop node of the secondary relay is different from that of the primary relay.

Condition (A) is applied to prevent routing loops in combination with the primary and the secondary relays. If packets are always forwarded to decrease the distance in *carry*, they will surely not create loops. Condition (B) is applied to prevent the same-street problem. Since RDV passes packets to the vehicles that comes from the previous-hop node that has the largest probability to visit the next-relay nodes, the vehicles from different previous-hop node are expected to go for different directions and visit different next-relays in many cases. One of the exceptions is when two roads are joining into a road, in which vehicles from those two source roads will go to the same direction. Excluding the exceptional cases, this rule solves the same-street problem with high probability.

**Figure 6:**
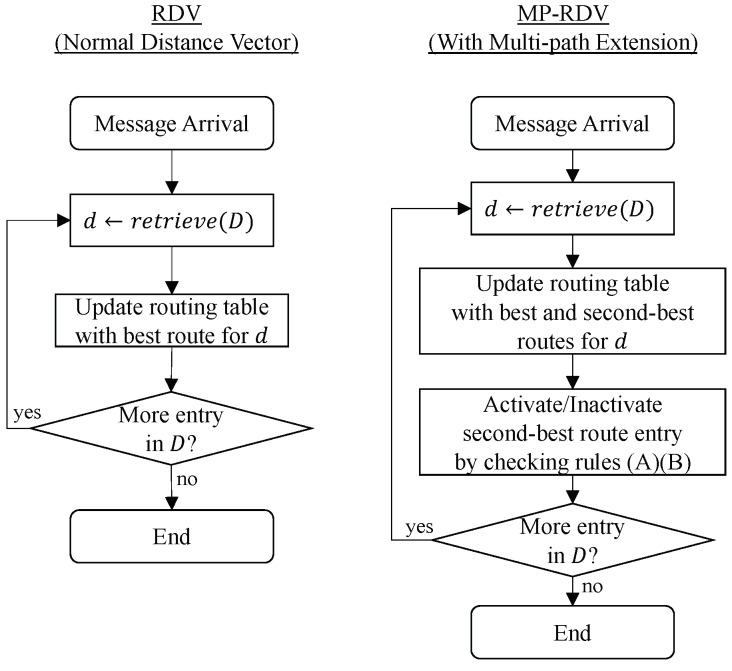
The flowcharts of routing-table updates in RDV and MP-RDV (Multi-Path RDV): MP-RDV maintains both the primary and secondary relays in the routing tables, and makes decisions on activating the secondary relay.

The flowcharts of RDV and MP-RDV procedures in updating routing tables are shown in Figure 6. In RDV, when a 1-carry or multi-carry message arrives, a node applies the distance-vector next-hop selection algorithm per destination d∈D, where *D* is a set of destinations included in the message. Specifically, for each *d*, a node compares the distance of old and new routes and updates the routing-table entry with the shorter one. In contrast, MP-RDV maintains the best and the second-best routes in the routing table. Namely, when a new entry for destination *d* comes with a message, a node compares the new route with two old routes, and updates the routing table entries with the best and the second-best ones. In case of MP-RDV, after this routing-table update, the node tests the second-best route whether it meets the above conditions (A)(B) or not. The second-best route is activated if both of them meet, and is inactivated otherwise.

### 4.3. Extending APD for Multiple Paths

The next problem in designing MP-RDV is how to distribute the duplicated copies of a packet into multiple paths while preserving the expected packet delivery ratio. This is solved by extending the APD mechanism of RDV to fit to the multi-path case.

Similar to the discussion of APD in RDV, let *P* be the preconfigured end-to-end expected delivery ratio to suffice. A node has two next-relays $n_{p}$ and $n_{s}$, which are the primary and the secondary ones. Let $p_{p}$ and $p_{s}$ be the corresponding paths to the destination, and we let the distance in carry of each be $\delta_{p}$ and $\delta_{s}$, respectively. Since MV-RDV forks delivery paths at every node in the forwarding paths, we further define variables related to the expected delivery ratio. Let $P_{x}$ be the expected delivery ratio from node *x* to the destination. Note that $P_{x}=P$ if *x* is the source node of the packet.

A node *x* about to forward a packet first computes the expected delivery ratio for each path to the destination. If *x* has two paths $p_{p}$ and $p_{s}$ to deliver packets, we let the expected delivery ratio for each of them be the same value $P_{x}^{\prime }$. To achieve the expected delivery ratio $P_{x}$ using those two paths, $P_{x}^{\prime }$ must hold the following equation:

(5) $$1-P_{x}={\left(1-P_{x}^{\prime }\right)}^{2},$$

which means that the packet loss probability from *x* to the destination is the same as the product of packet loss probability of two paths. From this, we obtain the following:

(6) $$P_{x}^{\prime }=1-\sqrt{1-P_{x}}.$$

By applying $P_{x}^{\prime }$ into the Formulas (1)–(3), we can obtain the number of duplicated packet copies $D_{l}$ required for each path $p_{p}$ and $p_{s}$ to suffice $P_{x}$.

**Figure 7:**
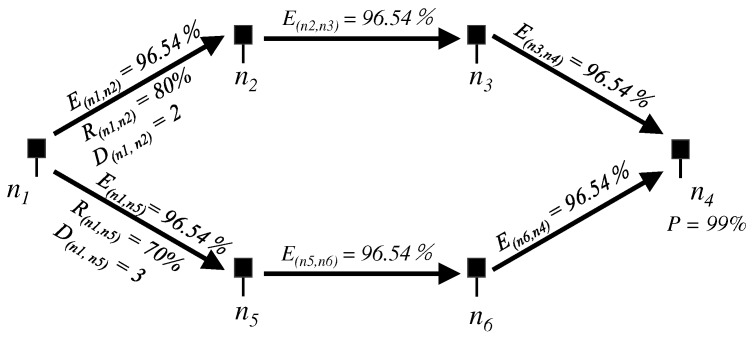
Example of ADP in MP-RDV: When two relays are available, nodes such as $n_{1}$ expect the same delivery ratio on both paths and computes the number of duplicated packets on each of them.

An example of APD in MP-RDV is shown in Figure 7. Suppose that a packet is generated at $n_{1}$ and destines $n_{4}$. Since $P_{1}=P=99\%$, we obtain the expected delivery ratio for each path from Formula (6) as $P_{1}^{\prime }=90\%$. From Formula (1), the expected delivery ratio $E_{l}$ for each link *l* in $p_{p}$ and $p_{s}$ is computed as 96.54%. Thus, from Formula (3), and provided that the delivery ratio estimated by SVS is $R_{\left(n_{1},n_{2}\right)}=80\%$, $n_{1}$ forwards $D_{\left(n_{1},n_{2}\right)}=2$ packets for the relay node $n_{2}$. Similarly, for $R_{\left(n_{1},n_{5}\right)}=70\%$, $n_{1}$ forwards $D_{\left(n_{1},n_{5}\right)}=3$ packets for $n_{5}$.

Note that the intermediate nodes such as $n_{2}$ and $n_{5}$ again fork paths. The required delivery ratio on $n_{2}$, which is $P_{2}$, is computed by the aggregation of expected delivery ratios in the primary path. Namely, in Figure 7, $P_{2}=E_{\left(n_{2},n_{3}\right)}E_{\left(n_{3},n_{4}\right)}=93.20\%$. Note that $E_{\left(n_{2},n_{3}\right)}$ and $E_{\left(n_{3},n_{4}\right)}$ take the same value and the value is notified by the parent node $n_{1}$ using the header space of data packets. This is formally written as follows. Let *x* be the parent node of *y* and $\delta_{p}$ be the distance in carry from *y* to the destination. Then, the expected delivery ratio on *y* is computed as $P_{y}=\delta_{p}E_{l}$, where $E_{l}$ is notified by *x*. Once the value $P_{y}$ is computed, *y* again distributes packet copies using multiple next-relays in the same process as described above.

### 4.4. Designing Delay Metrics for MP-RDV

To compare the performance of delivery delay metric with RDV’s built-in traffic-volume-based metric, we design the mechanism to apply the delay metric to MP-RDV. As a result, in MP-RDV, we can deploy either the traffic-volume metric or the delay metric described in this section. Same as the traffic-volume metric, the delay metric values are measured in cooperation with related nodes. The delivery delay on a path is defined as the sum of the transportation delays between nodes and the waiting delays on nodes on the path. The transportation delay is the time required for packets to travel among nodes, and the waiting delay is the time required for packets to wait on nodes until appropriate vehicles to pass the packet come. We designed the mechanism to collect those two sort of values and advertise them so that the delivery delay of any selected path is known in our distance-vector schemes.

Waiting delays are measured by *Hello* messages of RDV. We let $W(n_{a},n_{b})$ be the average waiting delay on $n_{b}$ for packets waiting for vehicles coming from $n_{a}$. Namely, $n_{a}$ is the previous-hop node for the packets’ destinations. In this case, node $n_{b}$ measures $W(n_{a},n_{b})$.

**Figure 8:**
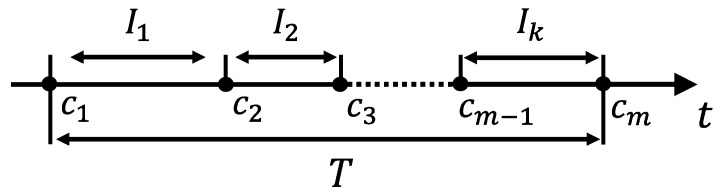
Illustration on measuring waiting delay: The waiting delay at a node is computed from the arriving time of *m* vehicles within the time period of *T*.

With Figure 8, we show the process to measure $W(n_{a},n_{b})$. Let $I_{i}\left(i=1,2,\cdots ,k\right)$ be the time difference of the two sequential vehicles that arrives at $n_{b}$ from $n_{a}$. Within the time interval *T*, the vehicles arriving at $n_{b}$ is denoted by $c_{i}\left(i=1,2,\cdots ,m\right)$ sequentially, i.e., the first vehicle in *T* arriving at $n_{b}$ is $c_{1}$, and the last one is $c_{m}$. We assume that the time on which packets arrive at $n_{b}$ follows a uniform distribution. Then, if a packet arrives at $n_{b}$ within the period of time *T*, the probability that it appears in $I_{i}$ is $\frac{I_{i}}{T}$, and it’s average waiting time is $\frac{I_{i}}{2}$. Thus, the average waiting time $W(n_{a},n_{b})$ is expressed as follows:

(7) $$W\left(n_{a},n_{b}\right)=\sum_{i=1}^{k}\frac{I_{i}}{T}\frac{I_{i}}{2}.$$

On the other hand, the transportation delay is measured through polling packets. Note that we define a new type of packets and pass a polling packet to every vehicle visiting the node. Every polling packet is carried to a neighbor node and the node gets to know the transportation delay between neighboring nodes. Note that *Hello* packets are not used for this purpose because the transportation process of RDV allows relaying packets among vehicles. Let $n_{a}$ be a neighbor of $n_{b}$. Then, when a vehicle visits $n_{a}$ and $n_{b}$ in this sequence, $n_{b}$ gets to know the transportation delay, denoted by $D(n_{a},n_{b})$, between those two nodes $n_{a}$ and $n_{b}$. The average transportation delay $\bar{D}\left(n_{a},n_{b}\right)$ in the period of time *T* is computed from all the delay values collected on $n_{b}$ in *T*.

If we assume time synchronization among every node, we can measure both the waiting and the transportation delays easily. However, even if we do not assume time synchronization among nodes, we can measure the waiting delay with clocks on stationary nodes, and the transportation delay with clocks on vehicles. Note that not so much accuracy is required to measure those delay values.

**Figure 9:**
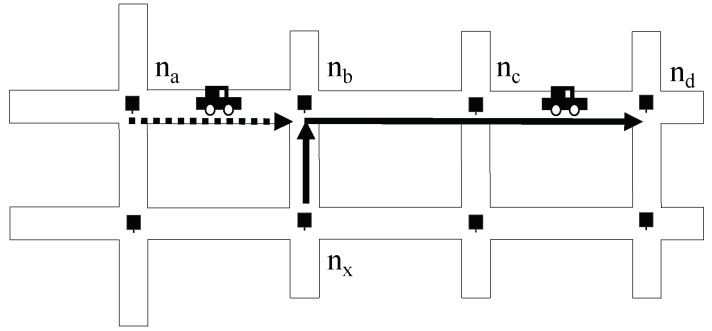
Illustration on computing delay metrics: The delay metric from $n_{b}$ to $n_{d}$ is computed as the sum of the waiting delay at $n_{b}$ and the transportation delays on $\left(n_{b},n_{c}\right)$ and $\left(n_{c},n_{d}\right)$.

The next step is to sum up those waiting and transportation delays as the routing metric values of links. See Figure 9. We expect a packet forwarded from $n_{x}$ is relayed by $n_{b}$ to reach $n_{d}$. When a vehicle moves from $n_{d}$ via $n_{c}$ to $n_{b}$, the measured average delays $\bar{D}\left(n_{b},n_{c}\right)$ and $\bar{D}\left(n_{c},n_{d}\right)$ are collected to $n_{b}$ by RDV messages. On the other hand, $n_{b}$ can compute the waiting delay for the previous node $n_{a}$, i.e., $W(n_{a},n_{b})$. Since the delay on link $\left(n_{b},n_{d}\right)$ is the sum of the waiting delay on $n_{b}$ (i.e., $W(n_{a},n_{b})$), and transportation delay $\bar{D}\left(n_{b},n_{c}\right)+\bar{D}\left(n_{c},n_{d}\right)$, the delay metric of link $\left(n_{b},n_{d}\right)$ corresponding to the previous-hop node $n_{a}$ is computed as

(8) $$M^{\left(dly\right)}\left(n_{a},n_{b},n_{d}\right)=W\left(n_{a},n_{b}\right)+\bar{D}\left(n_{b},n_{c}\right)+\bar{D}\left(n_{c},n_{d}\right).$$

This metric value is computed at $n_{b}$ and advertised to $n_{x}$ through RDV messages, and then $n_{x}$ is possible to forward packets to $n_{d}$ via a relay node $n_{b}$. In this way, the delay metrics of links are computed within the framework of RDV and advertised for being used in routing decision.

## 5. Evaluation

### 5.1. Methods

We evaluate the performance of MP-RDV through simulation and compared it with RDV and SADV. The main criteria in comparison is the basic communication performance measurement such as delivery ratio and delay under variations of vehicle density and data traffic amount. In addition, as a criterion for DTN routing, we measured the traffic load due to packet copies as well. We designed a scenario with the Kyoto city map in which we simulate the behavior of vehicles and packet delivery. We also measure the fault tolerance of MP-RDV under road failure; we designed a failure scenario in which roads are partially blocked, pretending a traffic accident occurred at the intersection.

We developed our own simulator with C++ language to simulate several routing protocols in relatively sparse vehicular networks. Because we evaluate the performance of MP-RDV in a sparse scenario in which networks behave as a DTN, we do not care about PHY and MAC protocols. Thus, in our simulation, we just assume that a certain amount of packet exchanges is possible when a vehicle meets another vehicle or a static node, as is usually assumed in evaluation of DTN schemes.

**Figure 10:**
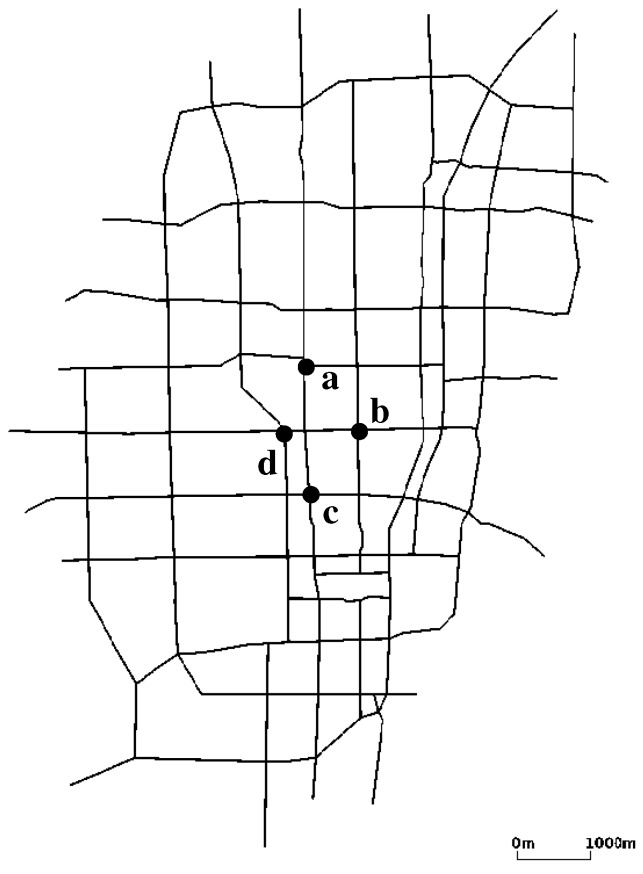
Kyoto City Map used in simulation.

We obtained a Kyoto roadmap data set in Open Street Map [28] and removed small streets from it to retrieve major roads as shown in Figure 10. In this map, we have 104 intersections in total, at all of which we place traffic signals and static nodes. As a result, static nodes are placed only at major intersections in the Kyoto city map. To generate vehicle traffic over the map, we used a traffic simulator SUMO [29]. The map includes 20 road ends, at all of which we generate vehicles with a constant time interval. We set the maximum speed of vehicles as 50 km/h. In SUMO, we adopt a probabilistic mobility model; in every intersection, 90% of vehicles go straight, and 5% turn right and left, respectively. If vehicles are not allowed to go straight at an intersection, 50% of them turn left and the rest right. Each vehicle has a buffer (i.e., a transmission queue) that holds 128 data packets. We assume that each static node has a transmission queue for each previous hop (remember that RDV distinguishes previous hops to select vehicles to pass packets), the size of which is 1280 packets each. Nodes and vehicles have the communication range 50 m, i.e., vehicles work as *intersection mode* if they are within the range of a static node, and as *road mode* otherwise.

In RDV and MP-RDV, every node updates its statistics and the routing tables every time the node exchanges messages with a vehicle. This assumption offers the most sensitive updates of the statistics and the routing table, while we can take choices of less frequent updates to reduce the computational load. We set the expected delivery ratio P=99%. As for other parameters of RDV and MP-RDV, for which detailed descriptions are seen in [13], we set values as follows: We set the capacity of multi-carry messages per vehicle $M_{MC}=20$ (i.e., a vehicle deletes a multi-carry message randomly when the loaded messages exceed this value). We set the maximum number of hops for a 1-carry node $N_{carry}=10$ (i.e., *1-carry* links must be within 10-hops long). We set the minimum expected packet delivery ratio to be a relay node $T_{SC}=70\%$ (i.e., 70% delivery ratio is required to establish *1-carry* links).

For SADV, as preliminary tests, we measured the average delay of each link, i.e., each pair of neighbor static nodes with 1-hop distance, and used the fixed values in the shortest-path computation. We set TTL as the length of the shortest path plus 4, which allows using at most 4-hop longer paths than the shortest one as detour routes. From the preliminary tests, we found that the performance such as delivery ratio significantly improves if this value is +4 compared to +2 and 0, and that the values larger than +4 does not improve the performance but increasing the load of packet copies.

We transmit data packets for 1000 s after a 3600-s wait to obtain stable routing tables. In the first scenario, in which we measure the communication performance, we generated 1000 packets during the simulation time between randomly-selected static node pairs with a constant time interval. We generate 3–8 vehicles per minute per road-end, which is a relatively sparse traffic state. Note that three vehicles per minute would be sparse enough to demonstrate the case of rural area or night-time scenarios. We take the average of 10 repetitions to show the results.

To consider the uncertainty of vehicle behavior under sparse deployment of static nodes, we make vehicles pretend to leave major roads for entering minor streets or parking lots along the road, and again join in to major roads. Because we do not have static nodes at minor intersections in the sparse scenario, we cannot trace a part of vehicles that escapes into minor streets, etc. Thus, we treat the uncertain behavior of vehicles as a probabilistic phenomenon. Specifically, we probabilistically select vehicles at each section of roads between two static nodes (i.e., at each links), and flush (i.e., delete) all packets and messages held on them. This *flush operation* pretends that a vehicle leaves the road and simultaneously another vehicle joins. We let the probability of flushing at each road section be 0%, 1.0%, and 2.5% to measure the effect of uncertainty of vehicle movement.

On the other hand, in the second scenario in which we measure the fault tolerance, we block the intersections a, b, c, and d shown in Figure 10 to pretend a traffic accident in the intersection. This traffic accident occurs at 500 s after the starting time of data transmission. With this scenario, we observe the degree of packet loss during the time period before converging to the new shortest paths set.

### 5.2. Results

**Figure 11:**
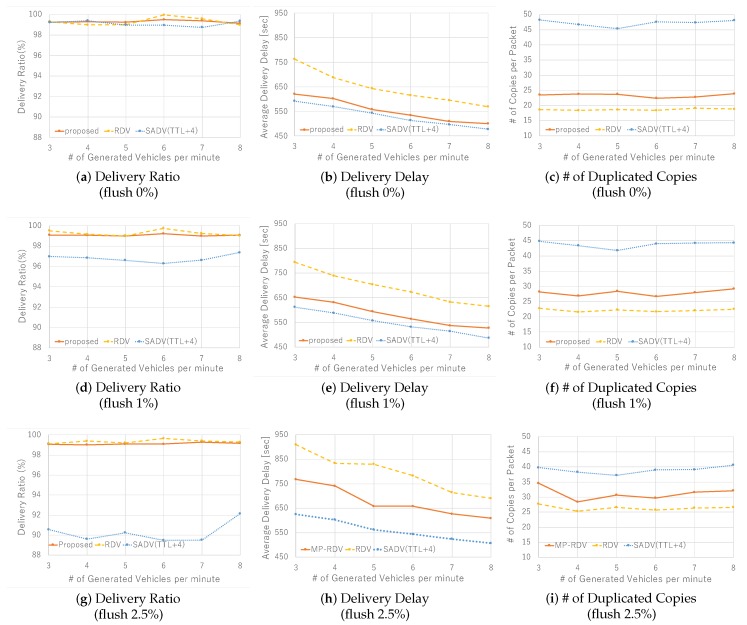
Performance with various vehicle density: delivery ratio, delivery delay, and the number of duplicated copies are measured for each flush rate 0%,1%, and 2.5%.

Figure 11 shows the results on basic communication performance. Figure 11a–c show the performance comparison results under the flush rate 0%. In Figure 11a, we see that delivery ratio is all good and comparable. Note that both RDV and MP-RDV exceed the predefined expected delivery ratio P=99%. In Figure 11b, we see the trend that delivery delay naturally reduces as vehicle density increases. Note that MP-RDV significantly improves delay compared to RDV, and the value is close to SADV. In Figure 11c, we see that the number of SADV’s packet copies is far larger than the others. Note that the difference between RDV and MP-RDV comes from the rounding error of the ceiling function shown in Formula (3). Those results clearly show the characteristics of the three schemes; all achieve a high delivery ratio while SADV requires a large amount of duplication load, RDV expenses delivery delay, and MP-RDV performs the best without degradation in neither of them.

As for the effect of flush events, i.e., the effect of uncertainty of vehicle behavior—see Figure 11d–i. The case of flush rate 1% is shown by Figure 11d–f. Although the trend is not so different from the case of 0%, we find that the delivery ratio of SADV is significantly degraded. This means that SADV is so sensitive to flush rate, whereas Adaptive Packet Duplication (APD) deployed in RDV and MP-RDV well works to keep the delivery ratio to 99%. The cases when the flush rate is as high as 2.5% are shown in Figure 11g–i. The same trend as 1% case is still kept, and RDV and MP-RDV still keep 99% delivery ratio. In Figure 11h, we see that the delay of RDV and MP-RDV is increased, meaning that RDV and MP-RDV is sensitive to flush rate on delay performance. In Figure 11i, the number of packet copies for RDV and MP-RDV increases, which is natural because they require larger number of packet copies when the packet loss ratio goes high. In contrast, SADV’s delivery ratio in Figure 11g is significantly degraded and the load due to packet copies in Figure 11i is reduced as well. This is because many packets are lost early before generating many packet copies, meaning that SADV is very weak against flush events. To summarize the effect of flush rate, we see that RDV and MP-RDV are able to keep the given delivery ratio 99% although delay and duplication load increases to some extent, whereas the performance of SADV significantly degrades due to packet loss.

**Figure 12:**
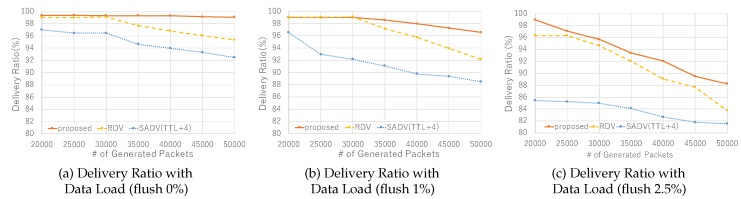
Delivery ratio with various data loads: delivery ratio under variation of data load is shown for each flush rate 0%,1%, and 2.5%.

Performance under variation of traffic load is shown in Figure 12. With flush rate 0%, we see that RDV fails to keep 99% delivery ratio when generating over 30,000 packets, whereas MP-RDV keeps 99% even at 50,000 packets. From this, we clearly conclude that MP-RDV has a larger traffic capacity due to the load balancing function over the multiple paths. In a 1% case, the same trend is seen although MP-RDV fails to keep 99% delivery at the point of 30,000 packets generation. In a 2.5% case, all fail to keep a high delivery ratio, while MP-RDV marks the best performance among them. In summary, the results show that MP-RDV has the largest network capacity among three, and has the highest ability to keep the expected delivery ratio.

**Figure 13:**
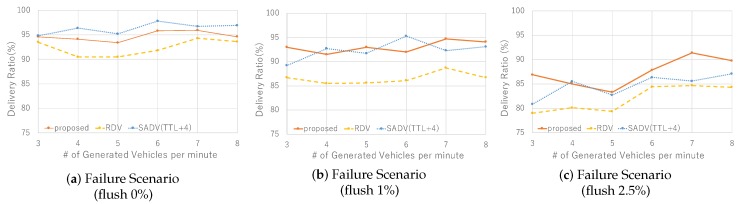
Performance in failure scenario: delivery ratio sensitively degrades as flush rate increases.

Finally, in Figure 13, we show the results on robustness against failure. We see that the performance of MP-RDV and SADV is better than RDV regardless of flush ratio, which is because multi-paths improve the robustness against failure. This means that the robustness of RDV against failure is improved with multi-path functionality.

### 5.3. Evaluating Delay Metrics

We compared the performance of two routing metrics, i.e., the traffic volume metric and the delivery delay metric, under the same evaluation procedure as described above. We fix the routing scheme to MP-RDV and see the effects of the routing metrics and flush ratio.

**Figure 14:**
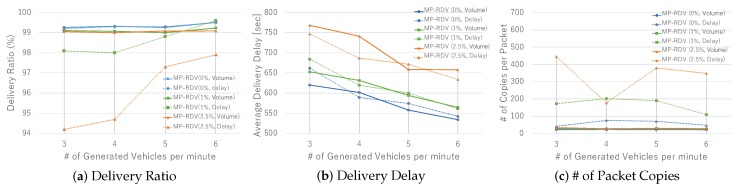
Comparing two routing metrics: delivery ratio, delivery delay, and the number of packet copies are compared with both delay and volume metrics.

Figure 14 shows the results. First, we focus on the case of 0%, and find that both traffic volume and delivery delay metrics have no difference in all of those three performance criteria. This is because traffic volume has relatively high correlation with delivery delay so that almost the same set of paths has been selected as a result.

Second, when we see the difference in flush ratio, we find that the delay metric significantly reduces its performance as flush ratio increases in delivery ratio shown in Figure 14a, whereas the volume metric remains at 99%. This trend is the same in the load of packet copies shown in Figure 14c. The reason why this occurs is that delay metric has a large fluctuation due to traffic signals and vehicle traffic state. Thus, statistically the delay metric is less stable than the volume metric especially when flush ratio increases, which impacts the performance. When routing metrics fluctuate in a large range, routing paths also change frequently. This causes instability of networks and then the delivery ratio as well as the load of copies get worse as shown in Figure 14a,c.

**Figure 15:**
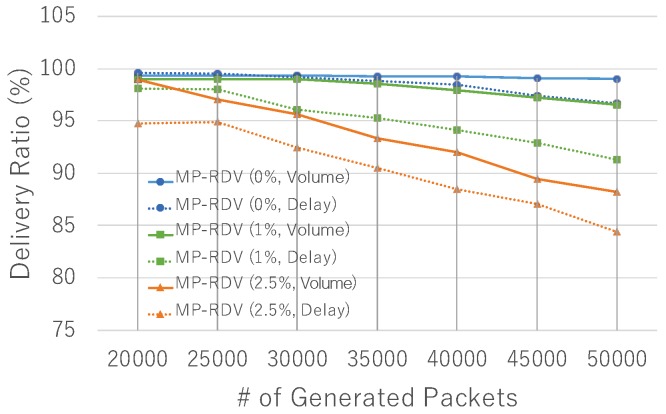
Metric Comparison under Variation on Data Rate.

**Figure 16:**
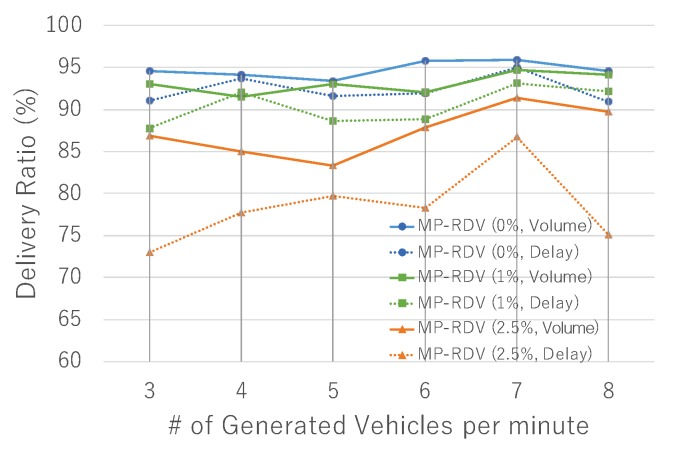
Metric Comparison in Failure Scenario.

In Figure 15, we show the performance under variation of generated data rate. The trend seen here is that performance gradually reduces as the data load increases, while performance of delay metric is lower than that of volume metric. This is also caused by the instability of the delay metric. Figure 16 shows the results of failure scenario, in which again the performance of the delay metric is lower than the volume metric.

## 6. Conclusions

We proposed two extensions on RDV to further improve the communication performance in static-node-assisted vehicular networks. From comparison with SADV, we extracted two points of potential improvement and designed them as an extension to RDV. The first extension is MP-RDV, a multi-path extension to RDV in which multiple paths are utilized for forwarding each of the packets to avoid load concentration on the shortest paths as well as to improve the delay performance. The second extension is the delay metric that measures the expected delivery delay as the sum of the waiting delay on nodes and the transportation delay between nodes. Our extensions inherit the characteristic of RDV to provide reliable communications that satisfies a pre-configured delivery ratio expectancy with a minimum packet duplication overhead. RDV achieves this in the following mechanisms. First, SVS measures statistics of vehicular movement at each static node located at major intersections to predict the outgoing direction of vehicles, and, second, APD controls the number of duplicated packets to achieve the required expected packet delivery ratio while minimizing the load of duplicated packets. The multi-path selection in MP-RDV cares for selecting two next-relays at each static node to distribute packets into the optimal paths while preventing routing loops. APD is also extended to handle multiple paths such that the load is well balanced among paths while guaranteeing preconfigured expected packet delivery ratio. The delay metric is also designed to work in the framework of RDV as well as MP-RDV. The waiting delay and the transportation delay are measured in cooperation with related nodes, and the measured metrics are advertised through the distance-vector messages in RDV.

Evaluation results show that MP-RDV has better performance in total than the conventional schemes considering the balance of several important measurements. MP-RDV is proven to have well-maintained delivery ratio, lower delay, high network capacity with load-balancing effect, and better fault tolerance performance. This shows that the multi-path strategy is beneficial in RDV schemes. On the other hand, the delay metric has been proven to have reduced performance compared to the traffic-volume metric that was proposed as a built-in metric in RDV. We found that, although those two metrics chose almost the same set of forwarding paths under stable vehicular traffic patterns, the delay metric is statistically unstable under fluctuation on traffic patterns coming from traffic signals, unforeseen driver’s behaviors, etc. Finally, we conclude that multi-path strategy with traffic-volume routing metric is the best choice for the RDV scheme.

In considering future work, we would note that this work is a pure theoretic framework that does not use any practically available observable data such as several sensors on vehicles, current location, and current moving directions. Enhancing the proposed framework utilizing those sensor data is one of the most interesting directions for the future. 

## Figures and Tables

**Table 1:** Notations on Adaptive Packet Duplication (APD).

Variable	Value
*P*	Preconfigured expected delivery ratio.
δ	Distance in carry for the destination.
$E_{l}$	Expected delivery ratio on link *l*.
$D_{l}$	Number of duplicated copies on link *l*.
$R_{l}$	delivery ratio of link *l* estimated by SVS.

**Table 2:** Routing Table of $n_{2}$ in Figure 3.

Dest.	Next,-Relay	Prev.	Dist.	Del. Ratio	Metric
⋮	⋮	⋮	⋮	⋮	⋮
$n_{7}$	$n_{5}$	$n_{1}$	2	72%	0.064
⋮	⋮	⋮	⋮	⋮	⋮

**Table 3:** Routing Table of $n_{5}$ in Figure 3.

Dest.	Next,-Relay	Prev.	Dist.	Del. Ratio	Metric
⋮	⋮	⋮	⋮	⋮	⋮
$n_{7}$	$n_{7}$	$n_{6}$	1	90%	0.028
⋮	⋮	⋮	⋮	⋮	⋮
